# Abnormal Control of Orbicularis Oculi Reflex Excitability in Multiple Sclerosis

**DOI:** 10.1371/journal.pone.0103897

**Published:** 2014-08-01

**Authors:** Christopher Cabib, Sara Llufriu, Eloy Martinez-Heras, Albert Saiz, Josep Valls-Solé

**Affiliations:** 1 EMG Unit, Neurology Department, Hospital Clinic, Barcelona, Spain, IDIBAPS (Institut d'Investigació Augustí Pi i Sunyer), Facultat de Medicina, University of Barcelona, Barcelona, Spain; 2 Center for Neuroimmunology, Neurology Department, Hospital Clínic, Barcelona, Spain, IDIBAPS (Institut d'Investigació August Pi i Sunyer), Universitat de Barcelona, Barcelona, Spain; Institute Biomedical Research August Pi Sunyer (IDIBAPS) - Hospital Clinic of Barcelona, Spain

## Abstract

Brain lesions in patients with multiple sclerosis may lead to abnormal excitability of brainstem reflex circuits because of impairment of descending control pathways. We hypothesized that such abnormality should show in the analysis of blink reflex responses in the form of asymmetries in response size. The study was done in 20 patients with relapsing-remitting multiple sclerosis and 12 matched healthy subjects. We identified first patients with latency abnormalities (AbLat). Then, we analyzed response size by calculating the R2c/R2 ratio to stimulation of either side and the mean area of the R2 responses obtained in the same side. Patients with significantly larger response size with respect to healthy subjects in at least one side were considered to have abnormal response excitability (AbEx). We also examined the blink reflex excitability recovery (BRER) and prepulse inhibition (BRIP) of either side in search for additional indices of asymmetry in response excitability. Neurophysiological data were correlated with MRI-determined brain lesion-load and volume. Eight patients were identified as AbLat (median Expanded Disability Status Scale–EDSS = 2.75) and 7 of them had ponto-medullary lesions. Nine patients were identified as AbEx (EDSS = 1.5) and only 2 of them, who also were AbLat, had ponto-medullary lesions. In AbEx patients, the abnormalities in response size were confined to one side, with a similar tendency in most variables (significantly asymmetric R1 amplitude, BRER index and BRIP percentage). AbEx patients had asymmetric distribution of hemispheral lesions, in contrast with the symmetric pattern observed in AbLat. The brainstem lesion load was significantly lower in AbEx than in AbLat patients (*p* = 0.04). Asymmetric abnormalities in blink reflex response excitability in patients with multiple sclerosis are associated with lesser disability and lower tissue loss than abnormalities in response latency. Testing response excitability could provide a reliable neurophysiological index of dysfunction in early stages of multiple sclerosis.

## Introduction

Although the available criteria for the diagnosis of multiple sclerosis do not require neurophysiological testing [1], many aspects of the disease can be shown mainly or solely using neurophysiological studies [2], [3], [4], [5], [6], [7], [8], [9], [10]. This is the case, for instance, with some forms of hyperactivity, such as the well-known facial myokymic discharges likely due to ectopic generation of activity in demyelinating perinuclear lesions [11], [12] and other neurophysiological manifestations of altered excitability in brain, brainstem or spinal cord [13], [14], [15]. Altered excitability may be due to focal axonal damage or to alterations in the descending inhibitory control. The latter may be reflected in the analysis of brainstem reflex circuits, known to be susceptible to dysfunctions in supranuclear structures such as in the basal ganglia [16], [17], [18] or in the cerebral cortex [19], [20], [21].

The most common brainstem reflex is the trigeminal blink reflex (TBR), mediated by trigemino-facial ponto-medullary-circuits [22], [23], [24], [25], [26], [27]. Median nerve electrical shocks can also induce blink reflexes [28], [29], i.e., the somatosensory blink reflex (SBR), which afferent circuit is different from that of the trigeminal input and, therefore, permits the assessment of non-trigeminal brainstem circuits that end up activating facial motoneurons [30]. The prevalence of TBR abnormalities in patients with multiple sclerosis varied between 26% and 78% in the pre-MRI era [31], [32], [33], [34], and ranged from 40% [35] to 91% [36] in the few studies performed in correlation with MRI data. However these observations were always based on assessment of response latency, while the relevance of abnormalities in response size has not been studied so far.

The size of a reflex response is derived from the number of motoneurons activated after synaptic input. Therefore, once other variables are controlled, response size would reflect the level of motoneuronal excitability. In the case of the TBR, the fact that the *orbicularis oculi* (OOc) responses are generated in both sides to a unilateral stimulus helps in the assessment of asymmetries in the modulatory control. The R2 ipsilateral to the stimulation side is usually larger than the contralateral (R2c) response, i.e., the R2c/R2 ratio is lower than 1 [37], [38], [39], [40]. Therefore, the finding of the opposite pattern, i.e., that the R2c is larger than the R2 (R2c/R2 ratio larger than 1), may indicate, in the appropriate context, an enhancement of motoneuron excitability in the side where the larger R2c response is recorded [41]. Other forms of reflex excitability assessment are the blink reflex excitability recovery (BRER), examined by using pairs of stimuli at variable inter-stimuli intervals [42], [43], and the blink reflex inhibition by a prepulse (BRIP), examined by measuring the inhibition caused in the R2 response of the TBR by a conditioning low intensity stimulus of any sensory modality [44], [45], [18], [46]. Abnormal BRER is usually indicative of altered excitability in trigemino-facial interneurons [47], [48], [40], whereas an abnormality in BRIP should indicate lack of inhibitory control of sensory inputs in subcortical circuits [49], [50], [18], [51].

We hypothesized that patients with multiple sclerosis should present with an abnormal brainstem reflex excitability due to deranged modulatory influences from supranuclear centers, and that because of the often random distribution of brain lesions, such excitability abnormalities will likely be asymmetric. Therefore, we applied a battery of tests to measure excitability of OOc reflex circuits of either side in a homogeneous cohort of patients with relapsing-remitting multiple sclerosis and examined the possible correlation of such dysfunction with MRI data.

## Materials and Methods

The study was carried out in 20 patients with confirmed diagnosis of relapsing-remitting multiple sclerosis [1], who were prospectively selected from the outpatient Clinic of the Hospital Clinic of Barcelona. They were recruited for the study if they were ambulatory, had low to moderate scores on the Expanded Disability Status Scale (EDSS, 0 to 6.0) [52], were under stable immunomodulatory treatment and relapse and steroid-free for at least one month prior to inclusion. They were 12 women (57%), and the majority were right-handed (18 patients, 86%). The mean age of the group was 37+/-7 years (range from 27 to 53 years) and their mean disease duration was 9.7 years (ranging from 2 to 21 years). We also studied 12 age and sex matched healthy subjects, who served as the control group. Their mean age was 36+/-8 years, ranging from 26 to 52 years. Seven (58%) were women. Ten (83%) were right-handed. The Clinical Research Ethics Committee of the Hospital Clinic of Barcelona approved the study and all participants gave written informed consent.

All patients underwent a complete neurological exam, including motor and sensory domains and paying special attention to eventual disorders of cranial nerves. Data gathered included the EDSS and Brainstem Function System to assess physical disability.

For the neurophysiological testing, subjects were lying relaxed on an examination bed, in a quiet and dimly lit room at ambient temperature. Healthy subjects and patients underwent the same neurophysiological tests. All tests were done with either a MYSTRO5Plus electromyography (Vickers Medical, Surrey, UK) or a KeyPoint Net Electromyograph (Alpine Medical Instruments, USA), set with the exact same parameters for recording and stimulation. A brain and brainstem magnetic resonance imaging study was done for a correlation between neurophysiological abnormalities and neuroimage lesions.

### TBR and SBR

We used surface silver/silver chloride 9-mm diameter recording electrodes, attached over the OOc in both sides with active electrode over the middle part of the lower eyelid and the reference at the lateral cantus of the eye. To examine the TBR, stimulating electrodes were attached over the supraorbital nerve, the active at the supraorbital fossa and the reference 3 cm apart on the frontal surface. The stimulus intensity was set to elicit a stable R2, usually about 4-6 times the sensory perception threshold. The stimulus duration was 0.2 ms. Interstimuli intervals were at least 10–20 seconds. The band pass frequency filter was set at 20–2000 Hz. We obtained 5 responses in both sides to unilateral single stimuli applied to either side. To examine the SBR, a surface bipolar stimulator was attached to the ventral aspect of the wrist over the median nerve and single stimuli were applied without warning, the stimulus intensity being high enough to induce a clear twitch in the thenar muscles. OOc responses were obtained ipsilaterally to the stimulus applied to either side.

Response latency was measured from stimulus artifact to onset. Response size was measured as peak to peak amplitude for the R1 and as area for the R2, R2c and SBR. Area was measured in µV x ms, using the automated system of the electromyograph. For the TBR, data from each individual were averaged to obtain the mean and SD values out of the 5 responses to each stimulus side. In the case of the SBR, though, because of its fast habituation [30], only the response to the first stimulus was used for measuring. In case of absent responses, we did not introduce latency values while we entered 0 for the area value.

### BRER and BRIP

BRER was assessed by applying pairs of supraorbital nerve stimuli (conditioning and test) of the same intensity as used for the TBR, at intervals of 100, 200, 300, 400, 500 and 600 ms. Prepulse inhibition was examined using electrical prepulses applied to the index finger 100 ms before a supraorbital nerve electrical stimulus [53], [46]. The prepulse was a low intensity stimulus (usually less than 2 times the sensory perception threshold), which was noticed but unable to generate reflex responses on its own. Both, BRER and BRIP were tested in three consecutive trials. Responses were recorded from the OOc to ipsilateral stimuli in both sides. In BRER, we measured the size of the R2 response to the test stimulus as a percentage of the R2 response to the conditioning stimulus and expressed this value as a function of the interstimulus interval to build the excitability recovery curve. In BRIP, we measured the area of the R2 response in trials with prepulse and expressed its value in percentage of the mean R2 area obtained in trials without prepulse.

### Neurophysiological data analysis

We first analyzed the blink reflex data on response latency to identify patients that could show coincidental MRI focal lesions affecting the brainstem pathways involved in these responses. After that, we analyzed the blink reflex data on response size to test the hypothesized abnormalities in brainstem reflex excitability.

#### Response latency

To establish whether or not unilateral response latency values for R1, R2, R2c and SBR were abnormal, we used reference normative values obtained from our healthy subjects, which did not differ from those previously published elsewhere from our own laboratory [54], [30]. For the TBR, the reference values were 12.8 ms for R1, 38 ms for R2 and 40 ms for R2c. We also considered abnormal inter-side differences larger than 1.5 ms for R1 and 5 ms for R2, as well as a difference between R2 and R2c larger than 8 ms. For the SBR, we only considered that the patient had an abnormal SBR when latencies were beyond the cut-off value for healthy subjects (66.7 ms) [30], or when the response was consistently absent in one side and present in the other. Patients who fulfilled any of the above criteria were identified as the group of patients with abnormalities in blink reflex response latency (AbLat), to differentiate them from patients with no latency abnormalities (no-AbLat). AbLat patients were further analyzed in accordance to the pre-defined patterns described by Aramideh *et al*. [55]: An afferent pattern was considered when a delayed latency or an absent response of both R2 and R2c was found to unilateral stimulation. An efferent pattern was considered when a delayed or absent R2 response was present in one side to stimulation of either side. A commisural pattern was considered when the R2c was significantly delayed or absent, with normal R2 responses. Finally, a mixed pattern was defined when any of the above conditions were combined or a significant delay was found only in the R1.

#### Response size and excitability tests

For the analysis of the OOc response size, we first calculated the R2c/R2 ratio by dividing the response area of the R2c by that of the R2 to unilateral stimuli for either side. Therefore, we obtained the R2c/R2 ratio for right and left sides in each subject. We also averaged the values of the R2 area obtained in the same OOc muscle by stimulation of either side, i.e., the value of the R2 response obtained to ipsilateral stimulation was averaged with the value of the R2c response obtained to contralateral stimulation. The inter-side differences for both, the R2c/R2 ratio and the R2 area, were calculated in each subject. Data from healthy subjects were used to determine the cut-off reference values (mean+/-2 SD) for the absolute values and the inter-side differences in the two measures of OOc response size.

**Figure 1:**
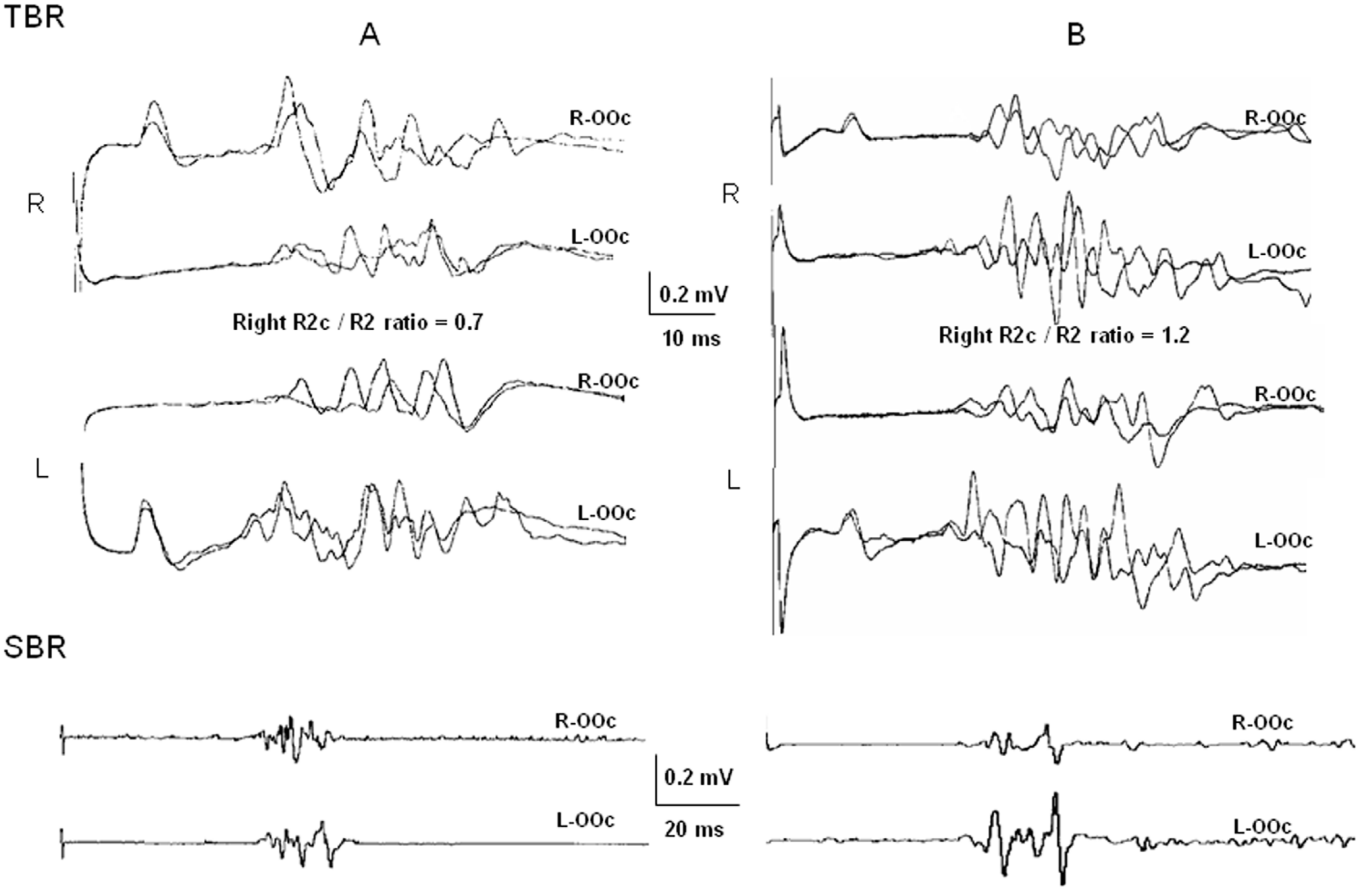
Blink reflexes in a multiple sclerosis patient showing a unilateral enhancement of excitability (AbEx). Recordings from bilateral *orbicularis oculi* (OOc; R = right and L = left) of the trigeminal (TBR) and the somatosensory blink reflexes (SBR). The graphs at the top of the figure (TBR) show both unilateral/early response (R1), and bilateral/late responses (R2 and R2c), to stimulation of the supraorbital nerve (SON) of either side (two traces are superimposed). The graphs at the bottom of the figure (SBR) show ipsilateral OOc responses to median nerve stimuli. The recordings of a healthy subject (**A**) are shown in the left side of the figure, and those corresponding to a representative patient with enhanced R2c/R2 ratio (AbEx, **B**) are shown in the right side. Note that the R2c is larger than the R2 to stimulation of the right SON in the patient but not in the healthy subject, i.e., the R2c/R2 ratio is below 1 in the healthy subject and above 1 in the patient. Note also the similar size of the SBR in responses of both sides in the healthy subject and the clear inter-side difference in the patient, who had a larger response in the left side (coincident asymmetry with the R2c/R2 ratio). Coincident enhanced unilateral OOc responses from both trigeminal and median nerve stimulation suggest left facial motoneuronal excitability enhancement.

We considered that patients who exceeded the normal cut-off limits, coinciding in the same side for both measures of response size, had abnormally enhanced blink reflex excitability in that side (AbEx). These were differentiated from the patients with no significant increase in OOc response size (no-AbEx). From the analysis of inter-side differences, we determined that the side with larger OOc response size had a relative excitability enhancement with respect to the side with smaller response size. Figure 1 shows an example of asymmetric OOc response size, compatible with a unilateral enhancement of excitability in the left side, taken from a representative patient.

In addition, we characterized further the abnormality by measuring the response size and the inter-side difference for the data gathered from the R1 and the SBR. Since the R1 and the SBR engage the activation of facial motoneurons by inputs from different sources, we considered that larger responses in the side with larger R2c would suggest a relevant contribution of enhanced motoneuronal excitability to the asymmetry in response size seen in AbEx patients.

We examined BRER separately by side in search for possible asymmetries in trigemino-facial reflex excitability. Then, we examined the possible correlation between the findings on BRER and the asymmetries in response size to determine whether there was a unilateral increase or decrease on brainstem excitability. For individual categorization of abnormalities, we considered abnormal the presence of a response to the test stimulus larger than 30% of the response to the conditioning stimulus at intervals of 300 ms or shorter [42], [67]. To simplify the data analysis in a single value suitable for group comparison, we just added up the percentage recovery at each interval for each subject to obtain the BRER index. We calculated the mean BRER index for each stimulation side for healthy subjects and patients. We considered that a BRER index abnormally larger in the side of the larger OOc response would mean that there was a relevant contribution of brainstem interneuronal excitability enhancement to the asymmetric response size seen in AbEx patients.

For BRIP, we also calculated the percentage inhibition separately for each side to evaluate somatosensory modulation of blink reflex excitability. We considered that an abnormally smaller BRIP percentage in the side of the larger OOc response would suggest a relevant contribution of an abnormal inhibitory effect of somatosensory inputs to account for the asymmetric response size seen in AbEx patients.

### MRI protocol and image analysis

A 3T Siemens Trio MRI scanner (Erlangen, Germany), with a 32 channel head coil, was used to obtain the following sequences: a) A 3D T1-weighted MPRAGE (Magnetization Prepared Rapid Acquisition Gradient Echo) sequence: Repetition Time (TR): 2050 ms, Echo Time (TE): 2.4 ms, Inversion recovery time (TI): 1050 ms, Flip angle: 9°, FOV: 220 mm. b) T2-weighted sequence are usually only viable option in 2D mode (3 mm slice of thickness), TR: 3200 ms, TE: 105 ms, Flip angle: 135 °, FOV: 250 mm and c) Sagittal 3D-FLAIR sequence with the same resolution of 3D T1-MPRAGE, TR:5000 ms, TE: 396 ms, TI: 1800 ms, FOV: 220 mm. In patients, a careful visual examination of the brainstem in all the sequences was performed to identify lesions in midbrain and particularly in the ponto-medullary area by a neurologist (SL) blinded to the results of the neurophysiological tests.

Individual white matter lesion masks were generated manually on the 3D T1-MPRAGE sequence using ITK-SNAP and the whole-brain lesion volume was obtained for each patient. The volumetric segmentation of supratentorial tissue and brainstem was performed on the T1-MPRAGE data sets using the Freesurfer image analysis suite (http://surfer.nmr.mgh.harvard.edu/). In patients, lesion refilling was used to avoid misclassification of white matter lesions and all the images were checked for errors and edited manually when necessary after the automatic segmentation processes. The complete topography of supratentorial tissue included all brain except cerebellum and brainstem, and the volume of each region was calculated and normalized for the intracranial volume. The supratentorial brain volume was obtained separately for each hemisphere, and we also obtained the whole brainstem volume. Subsequently, the volume of lesions in each side of the supratentorial tissue and in the brainstem was defined incorporating the lesion masks.

On the basis of supratentorial and brainstem lesion load and its distribution, a lesion probability map was created for each subgroup of patients. To do so, the lesion masks from each patient were normalized to the standard space using the FMRIB non-linear registration tool in FSL (Analysis Group, FMRIB, Oxford, UK). In the lesion probability map each voxel value can be thought of as an estimate of the probability that any subject of that subgroup has a lesion at that location.

### Statistical analysis

From the analysis of data, patients were separated in AbLat and no-AbLat, according to response latency, and in AbEx and no-AbEx, according to response size, for correlation with MRI findings. Parametric data were analyzed with one-way ANOVA. The 2X2 correlation test was used to examine differences in data distribution. Spearman's test was employed for correlations. Statistical significance was set on *p*<0.05. The Bonferroni's correction was used when necessary.

## Results

The median EDSS of the patients was 2.0 (range between 0 and 5.5), while the median on the Brainstem Funcional System score was 0.5 (range between 0 and 3.0). Eight patients (40%) had a past history of attacks affecting the trigeminal or facial nerves (Table 1). Neurological signs or symptoms of brainstem involvement were present in 4 patients (patient 1 with facial hypoesthesia, patient 5 with facial weakness, and patients 12 and 20 with facial myokymia). Myokymia was also present in patient 4, with no previous clinical history.

**Table 1:** Clinical, neuroimage and neurophysiological signs of brainstem involvement in all study patients.

Case^a^	Facial symptoms/signs		Brainstem MRI	Blink reflex (latency)	
	Past episodes	Current complaints	Lesion site	TBR	SBR
1	facial hyposthesia (R)	hyposthesia (R)	midbrain; pons; medulla	efferent (L)^b^	delayed (L)
2	none	none	pons; medulla	normal	normal
3	facial hyposthesia (L)	none	midbrain	afferent (L)^b^	normal
4	none	myokymia (R)	midbrain	normal	normal
5	facial weakness (R)	mild paresis (R)	midbrain; pons; medulla	commisural (R)^b^	normal
6	facial weakness (R)	none	midbrain	normal	absent (B)
7	none	none	none	normal	normal
8	none	none	midbrain; pons; medulla	afferent (R)^b^	absent (B)
9	facial hyposthesia (L)	none	none	normal	normal
10	none	none	midbrain; pons; medulla	afferent (L)^b^	delayed (B)
11	none	none	midbrain	normal	normal
12	facial weakness (L)	myokymia (L)	none	normal	normal
13	none	none	pons; medulla	mixed (B)^b^	delayed (B)
14	none	none	none	normal	normal
15	none	none	none	normal	normal
16	facial hyposthesia (B)	none	midbrain; pons; medulla	afferent (R)^b^	normal
17	none	none	none	normal	normal
18	none	none	pons; medulla	normal	normal
19	none	none	midbrain; pons; medulla	mixed (B)^b^	absent (B)
20	facial paresthesias (L)	myokymia (L)	midbrain; pons; medulla	normal	absent (B)

Abbreviations: TBR = trigeminal blink reflex; SBR = somatosensory blink reflex; R = right; L = left;

B = bilateral refer to the side of the involvement.

a Patient's identification number (in alphabetical order).

b Patients included in the AbLat group (see text for description of each TBR pattern).

### Response latency

Mean values for response latency obtained after pooling together all patients were within normal limits for the TBR (R1 = 12.5+/−2.5 ms; R2 = 35.5+/−4.9 ms, R2c = 37.2+/−5.1 ms) and for the SBR (55.6+/−8.7 ms). However, the analysis of individual TBR responses identified 8 AbLat and 12 no-AbLat patients, as listed in Table 1. Four patients had an afferent pattern, 1 had an efferent pattern, 1 had a commissural pattern and 2 had a mixed pattern. The SBR was delayed in three patients. The delay was limited to the left side in patient number 1, who had a left side efferent pattern in the TBR. It was bilateral in patient number 10, who had a left side afferent pattern, and patient number 13, who had a mixed pattern. The SBR was absent in 2 AbLat and in 2 no-AbLat patients. Four of the 8 AbLat patients have had previous attacks involving the trigemino-facial area (patients 1, 3, 5 and 16) and clinical signs remained in 2 of them (patients 1 and 5).

**Figure 2:**
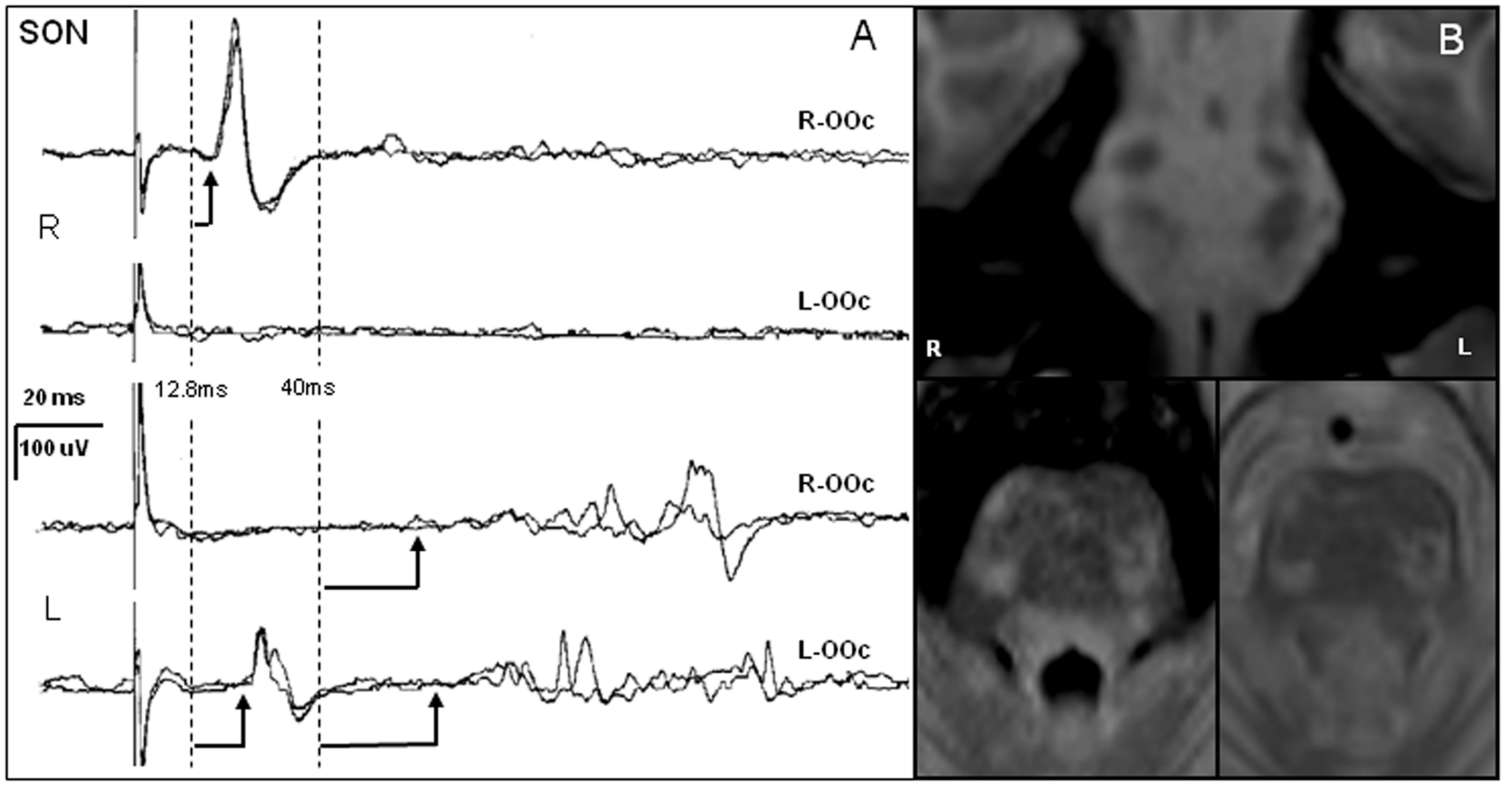
Trigeminal blink reflex and brainstem MRI of a patient with AbLat (n° 13). In **A** (left), *orbicularis oculi* (OOc) responses in both sides (R = right and L = left) are recorded after bilateral supraorbital nerve stimulation (SON, R = right, L = left; two traces are superimposed). Vertical dashed lines indicate the normal upper cut-off values for the latency of R1 and R2 responses and arrows indicate response onset. Note the delay in response latency for R1 in both sides and for the R2 and R2c to L-SON stimulation while these responses could not be obtained to R-SON (mixed pattern). In **B** (right), relevant MRI images from this patient are shown: coronal T1-MPRAGE (upper), axial FLAIR (lower left) and T2-weighted (lower right) images show evidence for multiple bilateral lesions in the pons.

The brain MRI showed brainstem lesions in all patients with AbLat, with hyperintense lesions on the T2-weighted sequence in the ponto-medullary area in 7 and in the midbrain in 7 patients as well (6 patients had lesions in both sites). The frequency of MRI lesions in the ponto-medullary area was significantly higher in AbLat patients (7 out of 8 patients; 87.5%) than in no-AbLat patients (3 out of 12 patients; 25%) (chi square, χ^2^; *p* = 0.006). Figure 2 shows neurophysiological recordings from patient number 13, who had a mixed pattern of abnormalities, and an MRI showing bilateral brainstem lesions located mainly in the pons tegmentum.

### Response size and excitability tests

#### R2c/R2 ratio and R2 area

The overall mean value of the R2c/R2 ratio was not significantly different between healthy subjects (0.815+/−0.093) and patients (0.809+/−0.302; ANOVA, *F* [1], [30] = 0.005, *p* = 0.947). No differences were either found between both groups for the overall R2 area mean value (4707.1+/−2864.3 uV x ms for healthy subjects and 5285.4+/−2767.2 uV x ms for patients; *F* [1], [30] = 0.638, *p* = 0.427).

**Figure 3:**
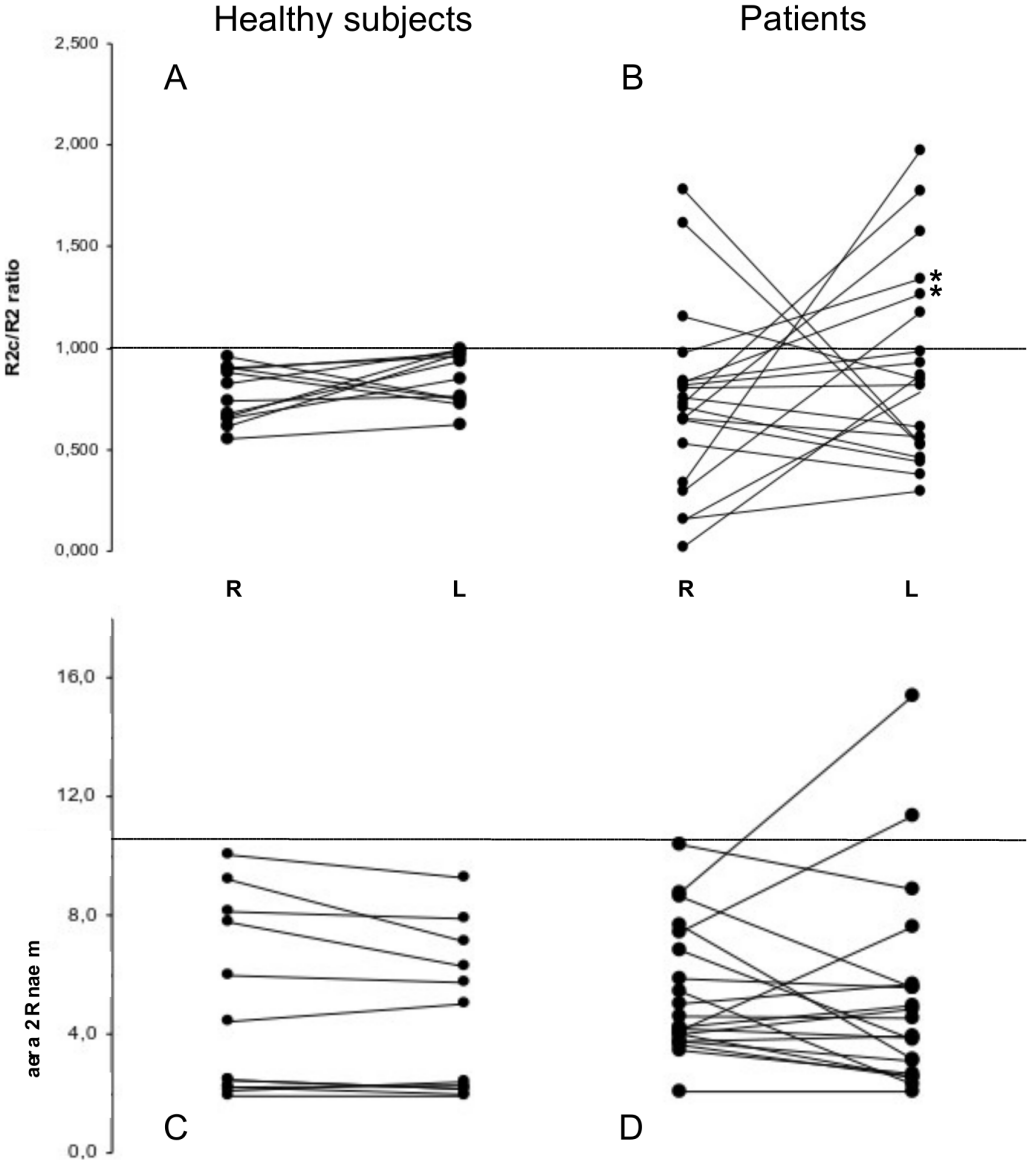
Graphical representation of values for right and left side R2c/R2 ratios (upper graphs) and mean R2 area (µV x s; lower graphs) in healthy subjects and patients with multiple sclerosis. The individual values for right (R) and left (L) sides are tied by a line to highlight the degree of between-sides asymmetry. Horizontal dashed lines indicate the normal upper cut-off values for both response size parameters. Note that healthy subjects show relatively more symmetrically distributed values with respect to patients for both the R2c/R2 ratio and the mean R2 area. A unilateral R2c/R2 ratio value above 1 was observed in 9 patients (lines reaching above 1 in panel **B**). Only 2 AbLat patients had a single value above 1 in the R2c/R2 ratio (marked with asterisks in **B**). Although no differences on the distribution of the mean R2 area values were apparent between both groups (lower graphs), 8 patients had a significant increase in the inter-side difference which was coincident with a unilateral enhancement on the R2c/R2 ratio (AbEx patients). These 8 subjects are identified in panel **D** because of their steeper slope in the inter-side.

Figure 3 shows the values for the R2c/R2 ratio and for the R2 area calculated on either side of patients and healthy subjects. In the great majority of healthy subjects (91.7%), a unilateral stimulus gave rise to a larger R2 than R2c response (R2c/R2 ratio lower than 1). No patient had a bilateral enhancement of the R2c/R2 ratio. In 9 patients, the R2c/R2 ratio exceeded the normal upper cut-off in one side (e.g. Fig.1B), showing larger inter-side differences than healthy subjects (Fig. 3B). Two of these 9 patients had also delayed OOc latencies (marked with an asterisk in the same figure).

Only two patients had a R2 area mean value exceeding the normal upper cut-off for one side only, while a significant increase in R2 area inter-side difference was found in 8 patients (Fig. 3D). Taking together the abnormalities in the R2c/R2 ratio and the R2 area, 9 out of 20 patients (45%) fulfilled the criteria for AbEx (all of them having an enhancement of the R2c/R2 ratio). From the remaining 11 no-AbEx patients, 2 exceeded the cut-off values only in one parameter (both being AbLat) and the other 9 patients had no abnormalities in the analysis of the response size.

#### R1 and SBR

Table 2 shows all data related to response size gathered from healthy subjects and AbEx and no-AbEx patients. The mean R1 amplitude values were not different for the three groups of subjects (ANOVA; *F* [2], [29] = 2.625, *p* = 0.09). However, there were significant differences in the mean R1 inter-side difference (*F* [2], [29] = 5.507, *p* = 0.01), which was found larger in AbEx patients than in healthy subjects in the *post-hoc* analyses (*p* = 0.008). In patients with AbEx, the R1 amplitude was statistically significantly larger in the side with larger OOc responses than in the contralateral side (paired t-test, *p* = 0.001). An inter-side difference value beyond the cut-off limits from healthy subjects was observed in 5 AbEx patients coinciding with the side of larger OOc response size.

**Table 2:** Data on response size calculated in healthy subjects and in patients categorized as AbEx and no-AbEx.

		R2c/R2 ratio	R2 area	R1 amp.	SBR area	BRER index	BRIP
Subjects			(µVx ms)	(µV)	(µVx ms)		(%)
HS	Mean	0.815	4707.1	165.1	1716.0	92.4	94.1
(n = 12)	(SD)	(0.093)	(2864.3)	(79.1)	(1061.1)	(33.7)	(4.7)
	Side-to-side Δ^a^	0.170	546.8	31.8	424.8	13.2	3.4
	(SD)	(0.107)	(616.7)	(29.7)	(410.5)	(7.2)	(3.0)
	Cut-off	0.384	1780.3	91.2	1245.8	27.6	9.4
AbEx	Mean	1.071**	6270.0	241.7	1628.0	84.8	73.3*
(n = 9)	(SD)	(0.149)	(3259.6)	(66.9)	(1839.4)	(66.8)	(28.4)
	Side-to-side Δ^a^	0.884**	2767.7**	111.2*	1148.0	33.6*	30.5**
	(SD)	(0.444)	(1934.1)	(63.7)	(1220.6)	(15.7)	(23.4)
no-AbEx	Mean	0.594*	4479.9	182.5	939.8	65.3	86.8
(n = 11)	(SD)	(0.207)	(2027.2)	(81.9)	(611.0)	(44.2)	(19.3)
	Side-to-side Δ^a^	0.249	1007.9	63.2	644.8	17.0	5.6
	(SD)	(0.254)	(1271.4)	(61.8)	(708.4)	(11.2)	(6.3)

Abbreviations: HS = healthy subjects; AbEx = patients with asymmetric response size and suspected abnormal excitability;

no-AbEx = patients that did not fulfill the criteria for AbEx; SBR = somatosensory blink reflex;

BRER = blink reflex excitability recovery; BRIP = blink reflex inhibition by a prepulse; R1 amp.  = amplitude of R1.

The individual's mean values (from right and left sides) on each response size measure were averaged for group comparison.

a Arithmetic difference between sides (right and left) on each response parameter. *Significant differences (*p*<0.05)

found in a subgroup of patients with respect to HS. ** Statistically significant differences found in AbEx patients with respect

to both HS and no-AbEx patients.

The SBR could not be elicited bilaterally in 2 out of the 12 healthy subjects (16.7%) and in 4 out of the 20 patients (20%), a non-significant difference (χ^2^, *p* = 0.9). The mean value of SBR area was not significantly different among the three groups (*F* [2], [29] = 1.295; *p* = 0.290). Similarly, the inter-side difference was not significantly different among groups (*F* [2], [29] = 2.006; *p* = 0.153). However, we still compared the SBR area between sides in the group of AbEx patients with a paired t-test, reaching close to significance (*p* = 0.063). An inter-side difference value beyond the cut-off limits from healthy subjects was found in 5 AbEx patients and in 2 no-AbEx patients.

#### BRER and BRIP

**Figure 4:**
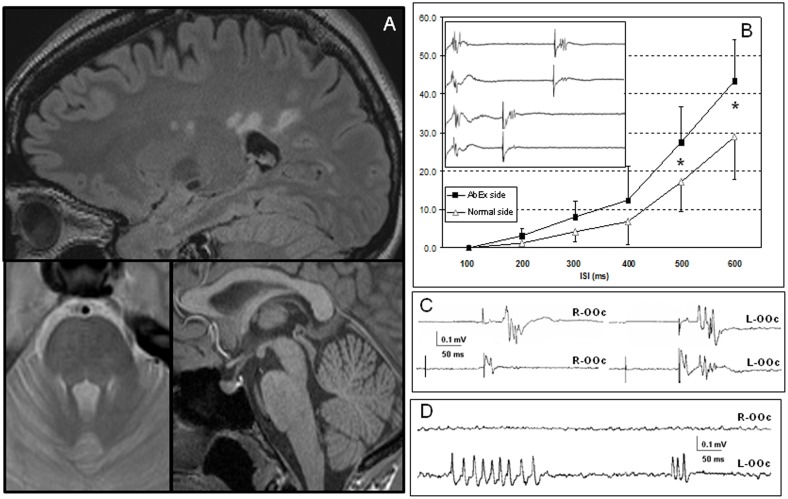
Blink reflex excitability abnormalities and brain MRI in a representative AbEx patient (n° 9). In **A**, supratentorial demyelinating lesions are identified (sagital FLAIR sequence, upper image) in absence of brainstem lesions (axial T2-weighted and sagital T1-MPRAGE sequences, lower images). In the inserted figure (**B**), significant individual differences on BRER can be observed between the side with largest OOc response size (AbEx side; first and third traces) and the contralateral side (normal side; second and forth traces) at the 600 (upper traces) and 300 miliseconds-ISIs (lower traces). In **C**, the BRIP was examined on both *orbicularis oculi* (OOc; R = right and L = left) to ipsilateral prepulses. Upper traces show the R1 and R2 responses obtained to ipsilateral supraorbital nerve (SON) stimulus without a prepulse. In lower traces, normal inhibition of the R2 response to a somatosensory prepulse delivered 100 ms before is only observed in the right side, but not in the left. In **D**, bilateral OOc surface recording in the same subject showing myokymic discharges on the left side. The BRER curve of all AbEx patients (n = 9) is also represented plotting the mean value (plus standard error) of each paired-stimuli interval (ISI, in miliseconds) (**B**). Data from both normal and AbEx sides (see above) were employed to build two curves. A higher mean value in the response's recovery was obtained in the side with largest OOc response size, with respect to the opposite side from 200 to 600 ms, with statistically significant differences in the 500 and 600-ISIs (marked with an asterisk, *p*<0.05).

Healthy subjects showed the expected inhibition of the R2 to the test stimulus at short interstimuli intervals, with a 4.4% (SD = 5.8%) recovery at the interval of 300 ms and 48.1% (SD = 20.1%) at the interval of 600 ms. There were no differences among groups for the mean BRER index values (Table 2; *F* [2], [29] = 0.873, *p* = 0.429), although two AbEx patients (number 1 and 6) showed enhanced BRER absolute values at the interval of 300 ms. However, inter-side differences on the BRER index fell beyond the cut-off reference values in 6 AbEx patients, who had relative enhancement of the BRER in the side where the OOc responses were larger. ANOVA showed a significant effect of group on inter-side BRER index difference (*F* [2], [29] = 8.536, *p* = 0.001), with significantly larger values in AbEx patients than in no-AbEx patients or healthy subjects (*post-hoc* Bonferroni test, *p* = 0.002). Figure 4 shows an example of BRER differences in one AbEx patient. Only one no-AbEx patient had an increase inter-side difference in the BRER index (number 13).

The mean percentage BRIP was significantly different among the three groups (*F* [2], [29] = 3.967, *p* = 0.030). The *post-hoc* analysis showed significantly lower BRIP in AbEx patients than in healthy subjects (*p* = 0.028), with no differences between healthy subjects and no-AbEx patients. There were also significant differences in inter-side BRIP values (*F* [2], [29] = 12.726, *p*<0.001), with a *post-hoc* analysis showing significantly larger values in AbEx patients than in healthy subjects and no-AbEx patients (*p*<0.001 for both comparisons). The lower cut-off value calculated from data in healthy subjects was 84.7%, which situated 10 patients out of the normal range, 7 of them being AbEx (e.g. Fig. 4).

#### Summary of inter-side differences

Inter-side differences coinciding with the asymmetry in the R2c/R2 ratio were present in 6 AbEx patients for R1 amplitude or SBR area, in 6 patients for BRER and in 5 patients for BRIP. Five AbEx patients had abnormalities in indices of asymmetry in 3 or more variables in addition to the enhanced R2c/R2 ratio. Only 1 AbEx patient (patient 19, who was also AbLat) had no abnormality other than the enhancement of the R2c/R2 ratio. The incidence of abnormalities in indices of response size asymmetry was significantly less in no-AbEx patients than in AbEx patients (χ^2^, *p* = 0.043).

### Differences between AbLat and AbEx patients in clinical and MRI findings

The median EDSS value for AbEx patients was lower than for AbLat patients (1.5; range 0–3.0 vs. 2.75; range 0–5.5). These differences were barely significant (ANOVA, *F* [1], [13] = 2.837, *p* = 0.049). The Braistem Funcional System score did not differ significantly between AbLat (median of 1.0, range 0–3.0) and AbEx patients (median of 0, range 0–1.0; *F* [1], [13] = 3.318, *p* = 0.092). Clinical signs of facial hyperactivity were referred by three patients (patients number 4, 12, and 20). Two of these patients were AbEx (4, 12) and none was AbLat. They all described tiny superficial periocular movements (i.e., myokymia) that, in 2 of them, showed as motor unit action potentials and bursts of interference pattern in surface EMG recordings. Repetitive short bursts of action potentials, resembling a myokymic discharge was indeed seen in patient number 9 who was also AbEx (Fig. 4D).

**Figure 5:**
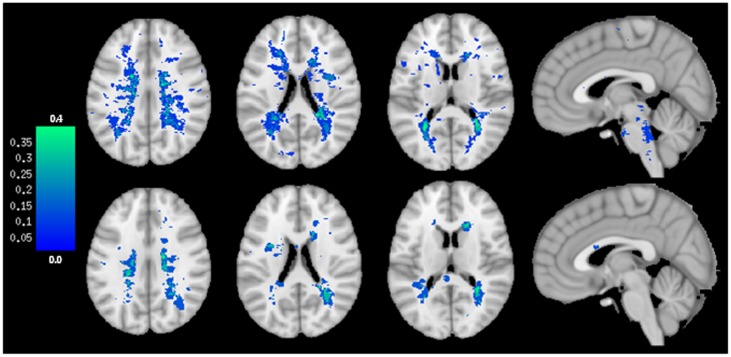
Lesion probability map in patients with AbLat (upper sequences) and AbEx (lower sequences). The distribution of lesions is shown at a supratentorial and brainstem level (representative sequences). In the probability map each voxel value (colored from deep blue to green) could be thought of as an estimate of the probability (from 0.0 to 0.4) that each subject of the subgroup had a lesion at that location. A different pattern of lesion distribution can be clearly distinguished between both groups.

In the MRI analysis, patients with AbLat had more frequent brainstem lesions than patients with AbEx (Table 1, Fig. 5). The two patients presenting with both, AbLat and AbEx (patients 1 and 19) showed ponto-medullary hyperintensity lesions, as did also 5 other AbLat patients while this was not the case in the remaining 7 AbEx patients. AbLat patients showed significantly higher brainstem lesion load than AbEx patients (*F* [1], [13] = 5.402, *p* = 0.040). Other comparisons between the two groups on MRI data did not reach statistical significance (Table 3). However, the map representing the probability of lesion distribution at a supratentorial level showed a diffuse and symmetric pattern in AbLat patients and an asymmetrical distribution of lesions in AbEx patients (Figure 5).

**Table 3:** Data on brain volume and brain lesion-load MRI-acquired in patients with multiple sclerosis separated by the abnormalities found on the TBR.

MRI data	All patients	AbLat	AbEx	P value
	(n = 20)	(n = 8)	(n = 7)	AbLat vs. AbEx
Brain volume^a^				
Total supratentorial (x10^5^)	13.49 (0.65)	13.11 (0.68)	13.77 (0.58)	0.087
Total brainstem (x10^4^)	2.64 (0.26)	2.57 (0.27)	2.82 (0.18)	0.074
Brain lesion-load^a^				
Total supratentorial (x10^3^)	6.22 (7.45)	9.31 (9.40)	2.29 (2.21)	0.103
Right supratentorial	2.75 (3.60)	4.26 (4.79)	0.85 (0.80)	0.115
Left supratentorial	3.47 (3.96)	5.05 (4.71)	1.43 (1.43)	0.099
Total brainstem (x10^2^)	1.23 (1.28)	1.52 (1.43)	0.15 (0.16)	0.040

Abbreviations: TBR = trigeminal blink reflex; AbLat = patients with delayed latencies on the TBR;

AbEx = patients with enhanced R2c/R2 ratio and no latency abnormalities.

a All data regarding to volume and lesion-load (expressed as mean and SD values in mm^3^) were normalized

to the individual brain size using a correction factor (VSCALING).

## Discussion

The typical neurophysiological observation in patients with multiple sclerosis is an altered conduction time in central sensory or motor tracts. This is the case with the somatosensory, auditory or visual evoked potentials, which may be delayed in correspondence with the presence of demyelinating lesions along the respective sensory paths [56], [57], [31], [4], [58]. We have indeed found a delay in latency of the OOc reflex responses to trigeminal stimuli, a finding already reported in the pre-MRI era [31], [33], [59], [34], which is likely to indicate focal lesions. However, the main finding in our study is the observation of abnormalities in the excitability of the trigemino-facial reflex circuits, an observation that has not been previously reported in multiple sclerosis. It is known, though, that a focal lesion in neural tissue may manifest in abnormal function of distant circuits and a change in reflex excitability might be a marker of such functional disorder [19], [21]. There are only a few published reports that show blink reflex abnormalities in patients with multiple sclerosis attributed to lesions at supranuclear level [36]. After our assessment of excitability in OOc responses, we identified a particular subpopulation of patients with enhanced excitability (AbEx) that, in comparison to patients with abnormalities in response latency (AbLat), were less disabled and had lower tissue loss, suggesting that this abnormality take place at earlier stages of the disease.

### AbLat patients

From the analysis of response latency, we identified a subgroup of 8 patients with delayed latency patterns (40% of patients in our study). In most instances, the pattern of latency abnormalities was afferent but other patterns were also found in single patients. Most AbLat patients (87.5%) had evidence of MRI demyelinating lesions affecting an area of the brainstem where the trigemino-facial reflex circuit has been located [22]. The only patient in whom there were no ponto-medullary lesions that could account for the delay on the blink reflex, had still a brainstem lesion located at the level of the midbrain. Similar frequencies of abnormal latency patterns and MRI-correlation with lesions of ponto-medullary localization in patients with multiple sclerosis have been previously reported by other authors [60], [61], [35]. The good correlation between MRI and neurophysiological findings points to an impairment in the normal neural conduction time through the TBR pathways caused directly by a lesion in some parts of the reflex arc. Taking the MRI results as the gold standard, the finding of latency abnormalities in the TBR would have an estimated sensitivity of 70% (7 positive abnormalities in the blink reflex out of 10 patients with positive MRI signs of ponto-medullary lesions) and an estimated specificity of 90% (9 negative observations out of 10 patients with no MRI signs of ponto-medullary lesions).

### AbEx patients

We considered that, apart from latency abnormalities, patients with multiple sclerosis could have other manifestations of dysfunction in their neurophysiological data. Indeed, we found that 9 patients had abnormally asymmetric response size on the TBR (mainly evidenced by examining the R2c/R2 ratio), together with corresponding asymmetries in other responses, indicating an abnormal unilateral enhancement of reflex excitability. On the basis of our results in various tests, we can suggest the most likely mechanisms accounting for the altered excitability: In 6 patients, the abnormality in the R2c/R2 ratio was accompanied by abnormalities in R1 or SBR amplitude; in also 6 patients it was accompanied by enhanced BRER index, and in 5 patients it was accompanied by reduced BRIP percentage. These results suggest, obviously, that more than one mechanism in each AbEx subject is likely contributing to the enhanced R2c/R2 ratio. The increase in R1 or SBR amplitude suggests that enhancement in facial motoneuronal excitability could be the cause of the asymmetric enhancement of the R2c/R2 ratio. Similar observations were reported in patients with hemifacial spasm who had either enhanced R1 [40] or more prevalent SBR [62] in the side of the spasm. The enhancement of BRER index indicates that lack of inhibition in trigemino-facial interneurones could be contributing to the asymmetric enhancement of the R2c/R2 ratio. A shift to the left in the BRER curve, as in the side with larger R2 area in our patients, has been reported in many supranuclear disorders such as Parkinson's disease [16], [17], [63], [43], dystonia [64], [65], [66], [67], [48], [68] and others. An enhancement of BRER can also be observed as a compensatory phenomenon occurring in the non-paretic side of patients with unilateral facial weakness [69], [70]. Reduced BRIP suggests lack of inhibitory control over sensory inputs as a mechanism leading to asymmetric enhancement of the R2c/R2 ratio. Prepulse inhibition refers to the inhibition caused by a low intensity stimulus of any modality that is unable to cause a recordable response by itself but induces significant changes in the response to a subsequent suprathreshold stimulus. Prepulse inhibition has proven to be effective in startle and TBR responses [53], [71]. In BRIP, as it was examined in our study, the inhibitory effect is seen on the R2 bilaterally when an inter-stimulus period beyond 70 ms is used. This effect is believed to be mediated by a circuit involving the pedunculopontine tegmental nucleus [72], [73], [74]. As far as we know, this is the first report on BRIP in patients with multiple sclerosis.

### Clinical implications

We considered that an asymmetry in the control of reflex excitability would be a likely feature of early stage multiple sclerosis. Asymmetries in response size are also relatively easy to detect in neurophysiological testing. This does not mean, though, that patients not showing asymmetric excitability enhancement do not have also an abnormal descending control of brainstem reflexes. Simply, the neurophysiological exams that we carried out, the ones with more acceptable clinical applicability, may not be able to show subtle changes in excitability. Although an abnormal asymmetry could, obviously, indicate decreased excitability in one side or increased excitability in the opposite side, the fact that some patients had features of facial hyperactivity in the form of myokymia or facial spasms suggest that the abnormality was the enhancement. Myokymia has been mostly attributed to pontine perinuclear lesions [12] but segmental demyelination away from the facial nucleus has also been demonstrated [75]. AbEx was found in only 2 patients of the 8 patients in the AbLat group in comparison to the 7 AbEx patients out of the 12 no-AbLat patients. This does not mean, though, that AbLat patients were less prone to have excitability disturbance. It is possible that lesions located caudally in the reflex arc might mask the effects of more rostral lesions of supranuclear localization in pathways that carry inputs directed to the facial motoneurons, and thus, it could explain the incapacity of neurophysiological studies on blink reflexes to reveal more often asymmetries in AbLat patients. Nevertheless, AbLat patients had significantly higher brainstem lesion load and nearly significant higher hemispheral lesion load and lower supratentorial and brainstem volumes than AbEx patients, and in fact all these data fits well with the significant higher EDSS score that we found in the former group of patients.

A limitation of this study is that our methods were focused on assessment of asymmetric alterations of excitability. We obtained indices of relative inter-side differences rather than absolute measures of abnormality. We cannot determine if the asymmetry found in excitability measures was due to an abnormal decrease in one side or an increase in the other side, although the presence of signs of hyperactivity in a few patients suggests enhancement rather than decrease. In any case, the novelty brought in by this study regards the abnormality in various indices of asymmetry of the OOc response size, which were larger in patients than in our control group of healthy subjects.

### Conclusions

Our results indicate that, in a number of multiple sclerosis patients with no trigemino-facial conduction impairment, the study of response size may show abnormalities in reflex excitability, based on the evaluation of indices of asymmetry. None of the patients with TBR excitability abnormalities showed MRI evidence of lesions in the circuits known to mediate the TBR. Instead, they had an asymmetrical distribution of the supratentorial lesions, and they represent a subgroup of less disabled patients with lower lesion load and lesser tissue loss than patients who present with abnormalities in trigemino-facial reflex latency. The findings of the present study suggest that neurophysiological testing may play an important role in the assessment of the brainstem excitability in patients with relapsing-remitting multiple sclerosis. Based on the findings of this study, we should consider multiple sclerosis as a disease manifesting not only by conduction abnormalities but also by excitability abnormalities, measurable with neurophysiological means. Abnormal unilateral OOc response excitability could be a reliable neurophysiological index of dysfunction in early stages of multiple sclerosis.
